# Believing processes around COVID-19 vaccination: An exploratory study investigating workers in the health sector

**DOI:** 10.3389/fpsyt.2022.993323

**Published:** 2022-09-21

**Authors:** Eva Fleischmann, Frederike Fellendorf, Elena M. Schönthaler, Melanie Lenger, Lena Hiendl, Nina Bonkat, Jolana Wagner-Skacel, Susanne Bengesser, Hans-Ferdinand Angel, Rüdiger J. Seitz, Eva Z. Reininghaus, Nina Dalkner

**Affiliations:** ^1^Department of Psychiatry and Psychotherapeutic Medicine, Medical University of Graz, Graz, Austria; ^2^Department of Medical Psychology and Psychotherapy, Medical University of Graz, Graz, Austria; ^3^Department of Catechetics and Religious Education, Karl-Franzens-University of Graz, Graz, Austria; ^4^Department of Neurology, Medical Faculty, Centre of Neurology and Neuropsychiatry, LVR-Klinikum Düsseldorf, Heinrich-Heine-University of Düsseldorf, Düsseldorf, Germany

**Keywords:** COVID-19 vaccination, workers in the health sector, cognition, emotion, credition, beliefs

## Abstract

**Background:**

The processes underlying believing have been labeled “creditions”, which are important brain functions between emotion and cognition. Creditions are influenced by both internal and external factors, one of which is the coronavirus disease 2019 (COVID-19) pandemic and the vaccination against the disease.

**Methods:**

To investigate believing processes shortly before the implementation of a mandatory vaccination in Austria, both vaccinated and unvaccinated workers in the health sector (WHS) were surveyed in December 2021/January 2022. In total, 1,062 vaccinated and 97 unvaccinated WHS (920 females) completed the online survey. Beliefs were assessed using the parameters of the credition model (narrative, certainty, emotion, and mightiness) with regard to (1) the COVID-19 pandemic in general, and (2) the vaccination. Type of emotion and narrative were divided into positive, negative, and indifferent. Moreover, the congruence between emotion and narrative was calculated.

**Results:**

The vaccination rate of the sample was 91.6%, with a significantly higher percentage of men being in the group of vaccinated (21.1%) as compared to unvaccinated individuals (12.4%). Pertaining beliefs about the COVID-19 vaccination, unvaccinated WHS reported more negative and less positive emotions as well as content of narrative than vaccinated WHS. In addition, they showed higher levels of certainty as well as mightiness while believing and felt less sufficiently informed about governmental and workplace-related COVID-19 measures. The groups did not differ in the type of emotion or content of narrative in their beliefs about the pandemic in general.

**Conclusion:**

In conclusion, unvaccinated WHS had more negative and less positive emotions and thoughts than vaccinated WHS in their beliefs about the COVID-19 vaccination and their motivations for not having received it. They were more certain about their beliefs and felt stronger negative emotions in their beliefs compared to vaccinated individuals. Providing unvaccinated WHS with adequate information might be helpful in reducing their mental burden.

## Introduction

Workers in the health sector (WHS) represent a group of particular interest at the heart of the coronavirus disease (COVID)-19 crisis, as they face additional stressors, such as a higher risk of infection (1), an intense additional workload (2), and problems arising from the long duration of wearing protective equipment (3). Symptoms of depression, anxiety, and post-traumatic stress disorder were reported by 20% (4) to 49% of WHS (5). Moreover, 38% of WHS suffered from insomnia during the pandemic, mainly due to working in a high-risk environment (6). The prevalence of COVID-19 infection was 7% when tested for the presence of antibodies and 11% when using polymerase chain reaction (PCR) in two separate meta-analyses (1, 7).

In Austria, the number of total infections increased from 1,175,785 on December 1, 2021, to 1,891,468 on January 31, 2022, and the number of deaths from or with COVID-19 increased from 12,458 to 13,669 (8, 9) (see Table 1). During this time, government measures to curb the spread of the virus included the obligation to wear masks in public and the obligation to be vaccinated or recovered from a COVID-19 infection when visiting public facilities, with the additional option of being tested for work (11). From November 15, 2021, to January 31, 2022, additional restrictions for unvaccinated people were introduced (12). A general lockdown was imposed from November 22 to December 11, 2021, mainly affecting the commercial and service sectors and private gatherings, while schools remained opened (13). See all COVID-19 restrictions between November 2021 and January 2022 in Austria on Table 2.

**Table 1:** COVID-19 cases, deaths and doses between November 2021 and January 2022 (10).

**Austria, up until 01/27/2022**
**Austria**	**Cases weekly**	**Deaths weekly**	**Doses weekly**
12/5/2021	55,195,000	408	722,745,000
12/12/2021	29,556,000	366	730,092,000
12/19/2021	21,607,000	317	679,382,000
12/26/2021	14,912,000	155	331,443,000
01/02/2022	20,957,000	118	253,943,000
01/09/2022	53,911,000	96	300,594,000
01/16/2022	104,168,000	72	347,250,000
01/23/2022	156,452,000	71	257,509,000
**Worldwide, up until 01/27/2022**
	**Total cases**	**Total deaths**	**Total vaccine doses**
Austria	1,727,661	14,042	17,448,173
United States	72,912,405	876,078	534,242,387
Worldwide	363,316,221	5,628,898	9,890,387,663

**Table 2:** COVID-19 restrictions in Austria between November 2021 and January 2022 (14, 15).

**Date**	**Restriction**
Since 11/15/2021	Lockdown for unvaccinated individuals
	Area-wide 2-G rules (Vaccinated/recovered)
	
12/17/2021	Vaccine for 12–17-year-olds and pregnant women
	Mandatory PCR-testing upon entering the country for those not vaccinated thrice
12/22/2021	70% of the Austrian population has an active vaccination certificate.
	73.6 % of people have been vaccinated at least once.
	38.5 % of people have already received a booster vaccination
01/10/2022	Tightening of the COVID-19 protective measures
	Outdoor mask requirement and retail 2-G controls are effective from January 11, 2022, to January 20, 2022, for now
	Lockdown for revaccinated people extended again until January 20, 2022
01/11/2022	Decision of a nationwide mandatory vaccination

At the beginning of December 2021, the decision to introduce compulsory vaccination in Austria was announced for the first time (16). Subsequently, a draft legislation to take effect on February 1, 2022, was presented by the governing parties (17). Shortly before Christmas, travel restrictions were imposed due to the Omicron variant (18). In January 2022, it was announced that starting at the beginning of February, the validity period of vaccination certificates would be reduced from 360 to 270 days, requiring many inhabitants to get vaccinated a third time (19). Compulsory vaccination was effective from February 5th, 2022, onwards, concerning, with some exceptions, adults aged 18 and older (20).

Vaccines licensed and at use in Austria were Vaxzevria (AstraZeneca), COVID-19-Vaccine Janssen (“Johnson & Johnson“), Comirnaty (BioNTech/Pfizer), and Spikevax (Moderna; 17). In total, 75.9% of Austrian citizens had been vaccinated once or more, 72.1% had received the second vaccine dose, and 49.8% had received the booster shot up until January 31, 2022 (14). In comparison, 63.3% of Europeans had received two vaccine doses (21).

Vaccination hesitancy has been noticed as a major global health threat (22). Vaccination hesitancy in WHS was found to range from 4.3% to 72% in the meta-analysis by Biswas et al. (23). Another meta-analysis showed that WHS displayed lower willingness to get vaccinated than the general population (24). The main characteristics of those wanting to get vaccinated were high education (23, 24), male gender (23, 25, 26), a history of previous influenza vaccination (24, 25, 27), and trust in the government (24). Relevant influence factors increasing the motivation to get vaccinated were confidence in the vaccine's safety and benefits (28), high perceived susceptibility to COVID-19 (29), and the desire to protect oneself and close ones from the virus (30). The leading concerns of WHS contributing to their hesitancy were related to safety, efficacy (23, 31), and potential side effects of the vaccination (23, 32). Furthermore, lack of information concerning the vaccination (32), distrust in the healthcare system, and concerns about the fast development of the vaccine are notable as well (33, 34). The effect of the exposure to patients with COVID-19 on vaccination hesitancy is unclear (35), although results of a systematic review point toward a decrease thereof (23).

Social influence factors should be mentioned as well: negative reports found on social media (36), low confidence in healthcare authorities (37), and distrust toward vaccines in individuals' social network contributed to vaccination hesitancy (34), while acceptance was increased by the need to conform to social norms (38, 39). Furthermore, feeling pressured by their employer to get vaccinated increased distrust and was associated with a higher number of declined vaccinations (40). In summary, there seem to be many different attitudes and beliefs for and against vaccination. However, studies that examine believing processes in more detail are still lacking.

Believing is a cognitive process that consists of formation, revision, and evaluation of beliefs (41, 42). Constructed on the basis of previous experiences (43) and influenced by internal and external factors, believing is hypothesized to be the result of perceptual and affective information processing (44). Beliefs are stable, but modifiable (45), can be changed if disproven (44), and allow predictions of future behavior (43). Neural correlates of believing have been found, cementing the formerly doubted existence of believing processes (46–48). The resulting concept of creditions (from the Latin *credere* = to believe) represents a dynamic process that can influence states of belief and further thinking, feeling, and acting (49).

The credition model comprises four main characteristics: proposition, certainty, emotion, and mightiness. “Proposition” refers to the content of the statement. “Certainty” represents the person's proclivity to believe the proposition. “Emotion” reflects the affective valence of the proposition. “Mightiness” refers to the degree of importance of the proposition (41).

The model of credition, scarcely explored in the clinical setting, gains new importance in the light of the pandemic, specifically when it comes to vaccination intentions. Although several studies have analyzed the acceptability of COVID-19 vaccination in WHS and its correlates, little is known about underlying reasons and possible believing processes that precede the decision to vaccinate or not. To fill this knowledge gap and learn more about the process of believing, this study aimed to analyze individual beliefs and connected credition parameters related to WHS's COVID-19 vaccination.

## Methods

### Procedure

This study was part of a large survey entitled “Psychosocial interests in the SARS-CoV-2 pandemic among healthcare workers in Austria” measuring resilience and stress factors in WHS during the pandemic. Inclusion criteria were voluntary participation (informed consent on the first page of the online survey), practicing a healthcare profession in Austria, and availability of a business e-mail address. Individuals were excluded if they were not in an active employment relationship in healthcare, refused participation after having received the e-mail, or did not complete the questionnaire. The study was conducted in accordance with the Declaration of Helsinki and approved by the Ethics Committee of the Medical University of Graz (EK number: 32 329 ex 19/20).

### Participants and materials

The online link was sent out to works councils, clinic management, professional associations, and healthcare facilities via e-mail to inform WHS to participate in the online survey. The study took place from December 16, 2021, to January 21, 2022, and was conducted using the software LimeSurvey (50). In sum, 2,321 WHS responded to the survey and 1,159 complete credition data sets were available (79.4% females).

To test individual beliefs, the Believing Questionnaire (BQ) was used. It was developed by an expert panel consisting of ND and JWS of the Medical University of Graz, who created the BQ, as well as HFA and RS, who were important advisors. Based on the BQ, the following two items were included in the survey:

Item 1: COVID-19 beliefs: *When I think about the coronavirus (COVID-19), I believe that (proposition)…*

Item 2: Vaccination/Non-vaccination motive beliefs: *I am vaccinated/not vaccinated against COVID-19, because I believe that (proposition)…*

In addition to the proposition, certainty [*On a scale from 0 (not sure) to 100 (very sure), how sure are you about your belief?*], emotion using an Emotion Wheel [*Please name an emotion that best describes your state while you are believing*], and mightiness [*On a scale from 0 (not at all) to 100 (very much), how strongly do you experience the emotion while believing?*] were assessed. Certainty and mightiness were metric variables, and emotion was categorized into positive (happy), negative (sad, angry, anxious, disgusted), and indifferent (surprised) emotions. In addition, it was evaluated whether the narrative was positive or negative and whether it matched the emotion (congruent) or not (incongruent).

### Statistics

Chi-square tests as well as two-tailed Fisher's exact tests were calculated to test for differences in sociodemographic variables (age, sex, positive COVID-19 tests, work area, mode of employment, and feeling informed about COVID-19-related measures) between vaccinated vs. unvaccinated WHS. Differences in content of narrative (positive, negative, indifferent), type of emotion (positive = happy, negative = disgusted, sad, anxious, angry, indifferent = surprised), and congruence between both variables (yes vs. no) were calculated using chi-square tests. As the assumptions for multiple analysis of variance (MANOVA) were not fulfilled, *t*-tests were used to compare vaccinated and unvaccinated WHS in the credition variables mightiness and certainty. All data were analyzed using IBM SPSS Statistics 27 and qualitative data were analyzed with MAXQDA qualitative analysis software. Word clouds as visual representation of word frequency of both items were created with MAXQDA.

## Results

The sample consisted of 1,159 individuals, 1,062 (91.6%) of whom were vaccinated, i.e., had received at least one vaccine dose (see Table 3). Both groups comprised mostly full-time workers aged 18 to 70 years. They did not differ in age or mode of employment, however, there were sex differences: there was a higher percentage of vaccinated than unvaccinated men, as opposed to women. Unvaccinated WHS felt less sufficiently informed about governmental and workplace-related COVID-19 measures than vaccinated WHS, who felt more sufficiently informed about governmental measures.

**Table 3:** Sociodemographic characteristics and differences between vaccinated and unvaccinated individuals (*n* = 1,159).

**Variables (*n*, %)**	**Group**		**Test statistic**	***p* value**
	**Vaccinated**^**a**^ **(*****n*** **=** **1,062)**	**Unvaccinated (*****n*** **=** **97)**		
Age			χ^2^4 = 3.20	0.525
18–30	293 (27.6%)	28 (28.9%)		
31–40	271 (25.5%)	30 (20.9%)		
41–50	259 (24.4%)	24 (24.7%)		
51–60	215 (20.2%)	14 (14.4%)		
61–70	24 (2.3%)	1 (1.0%)		
Sex			χ^2^1 = 6.87^b^	**0.039**
Female	836 (78.7%)	84 (86.6%)		
Male	224 (21.1%)	12 (12.4%)		
Other	2 (0.2%)	1 (1.0%)		
Having been tested positive for COVID-19			χ^2^(1) = 17.41	**< 0.001**
Yes	191 (18.0%)	35 (36.1%)		
No	871 (82.0%)	62 (63.9%)		
Working area			χ^2^(1) = 0.00	1.000
Clinical Work	970 (91.3%)	89 (91.8%)		
Administration	92 (8.7%)	8 (8.2%)		
Mode of employment			*χ^2^*(1) = 1.20	0.368
Full-time	757 (71.3%)	65 (67.0%)		
Part-time	295 (27.8%)	32 (33.0%)		
Marginal	10 (0.9%)	0 (0.0%)		
Feeling informed about new governmental measures^c^			χ^2^(4) = 72.58	**< 0.001**
Not correct at all	49 (4.6%)	23 (23.7%)		
Not rather correct	175 (16.5%)	28 (28.9%)		
Partially correct	339 (31.9%)	23 (23.7%)		
Rather correct	392 (36.9%)	18 (18.6%)		
Fully correct	107 (10.1%)	5 (5.2%)		
Feeling informed about new workplace-related measures^d^			χ^2^(4) = 24.28	**< 0.001**
Not correct at all	46 (4.3%)	14 (14.4%)		
Not rather correct	151 (14.2%)	21 (21.6%)		
Partially correct	285 (26.8%)	20 (20.6%)		
Rather correct	420 (39.5%)	32 (33%)		

^a^At least one shot; ^b^Two-tailed Fisher's exact test was used; ^c^“Do you feel sufficiently informed about the adoption of new governmental measures concerning the current COVID-19 pandemic?”; ^d^“Do you feel sufficiently informed about the adoption of new workplace-related measures concerning the current COVID-19 pandemic?”. Values in bold indicate statistically significant results.

**Figure 1:**
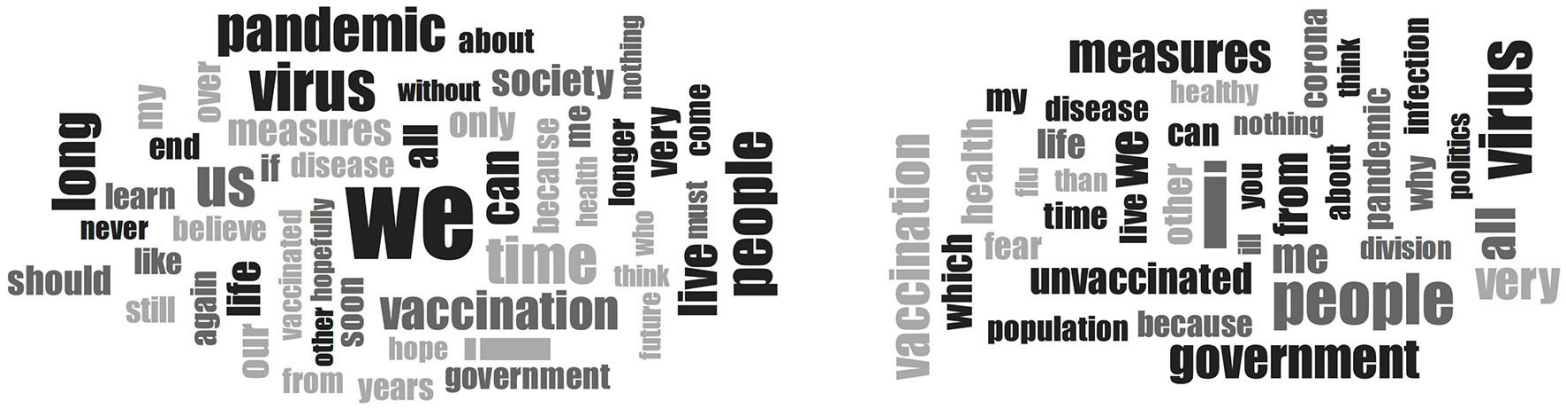
Word clouds of item 1 (“When I think about the COVID-19 pandemic, I believe”) of vaccinated vs. unvaccinated workers in the healthcare sector.

Regarding the COVID-19 pandemic in general (item 1), chi-square tests showed no differences in content of narratives, emotions, or congruence (see Table 4). However, *t*-tests found that unvaccinated individuals reported higher levels of both certainty and mightiness in their beliefs about the COVID-19 pandemic. Figure 1 displays the word clouds of vaccinated vs. unvaccinated individuals, and Table 5 shows the frequencies of the most commonly used words (In vaccinated individuals, the most frequent word was “We” and in unvaccinated “I”).

**Table 4:** Descriptive statistics of the believing parameters of vaccinated and unvaccinated workers in the healthcare sector (*n* = 1,159).

**Variables**	**Group**		**Test statistic**	***p*-value**	**Cohen's *d***
	Vaccinated^a^ (*n* = 1,062)	Unvaccinated (*n* = 97)			
**COVID-19 pandemic in general** ^**a**^				
Narratives (*n*, %)			*χ^2^*(2) = 3.26	0.196	
Positive	237 (22.3%)	14 (14.4%)			
Negative	735 (69.2%)	74 (76.3%)			
Indifferent	90 (8.5%)	9 (9.3%)			
Emotions (*n*, %)			χ^2^(2) = 5.68	0.058	
Positive	171 (16.1%)	8 (7.2%)			
Negative	751 (70.7%)	74 (76.3%)			
Indifferent	140 (13.2%)	16 (16.5%)			
Congruence^b^ (*n*, %)			χ^2^(1) = 3.09	0.079	
Congruent	918 (86.4%)	77 (79.4%)			
Incongruent	144 (13.6%)	20 (20.6%)			
Certainty^c^ (*M* ±*S)*	83.45 (19.26)	89.26 (16.21)	*t* (122.158) = 3.32	**0.001**	0.31
Mightiness^c^ (*M* ±*SD)*	69.78 (24.22)	77.74 (25.15)	*t* (1,157) = 3.40	**< 0.001**	0.36
**Vaccination** ^**d**^				
Narratives (*n*, %)			χ^2^(2) = 35.88	**< 0.001**	
Positive	608 (57.3%)	27 (27.8%)			
Negative	377 (35.5%)	64 (66.0%)			
Indifferent	77 (7.3%)	6 (6.2%)			
Emotions (*n*, %)			χ^2^(2) = 305.85	**< 0.001**	
Positive	944 (88.9%)	19 (19.6%)			
Negative	103 (9.7%)	71 (73.2%)			
Indifferent	15 (1.4%)	7 (7.2%)			
Congruence^b^ (*n*, %)			χ^2^(1) = 0.37	0.468	
Congruent	774 (72.9%)	74 (76.3%)			
Incongruent	288 (27.1%)	23 (23.7%)			
Certainty^c^ (*M* ±*SD)*	86.91 (17.69)	94.89 (9.69)	*t* (162.049) = 7.03	**< 0.001**	0.46
Mightiness^c^ (*M* ±*SD)*	76.73 (22.28)	86.41 (20.40)	*t* (117.930) = 4.44	**< 0.001**	0.44

^a^“When I think about the coronavirus (COVID-19), I believe that (proposition)”; ^b^Congruence between the narratives and the emotions; ^c^in percent; ^d^“I am vaccinated/not vaccinated against COVID-19, because I believe that (proposition)”. Values in bold indicate statistically significant results.

**Table 5:** Word frequencies of the 25 most frequent words for item 1 (“When I think about the COVID-19 pandemic, I believe”) in the Believing Questionnaire for the two groups.

**Vaccinated**			**Unvaccinated**		
Word	Frequency	%^a^	Word	Frequency	%^a^
We	314	2.63	I	92	2.69
I	313	2.63	Virus	52	1.52
Virus	158	1.33	People	48	1.40
Time	151	1.27	All	36	1.05
Us	148	1.24	Government	36	1.05
People	146	1.22	Vaccination	36	1.05
Pandemic	135	1.13	Measures	34	0.99
Long	131	1.10	We	34	0.99
Vaccination	115	0.96	From	28	0.82
Can	114	0.96	Me	28	0.82
Live	112	0.94	Health	26	0.76
All	97	0.81	Unvaccinated	26	0.76
Life	88	0.74	Which	24	0.70
Measures	82	0.69	COVID	22	0.64
Our	80	0.67	Life	22	0.64
Society	80	0.67	Other	22	0.64
My	69	0.58	Can	20	0.58
Over	66	0.55	Corona	20	0.58
Soon	65	0.55	Fear	20	0.58
Again	60	0.50	Live	20	0.58
Learn	60	0.50	My	20	0.58
Would	60	0.50	Pandemic	20	0.58
Me	59	0.50	Time	20	0.58
Still	59	0.50	Why	20	0.58

^a^Shows what percentage of the total words the word represents.

**Figure 2:**
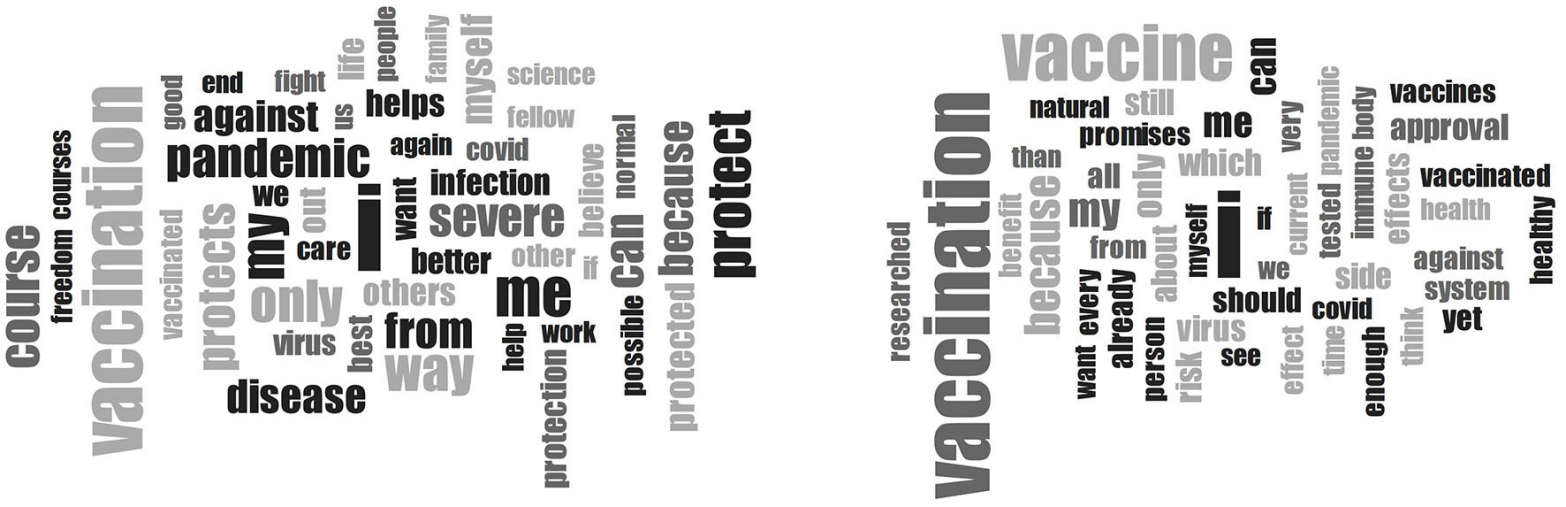
Word clouds of item 2 (“When I think about the COVID-19 vaccination, I believe”) of vaccinated vs. unvaccinated workers in the healthcare sector.

When believing about the motives for vaccinating or not vaccinating (item 2), chi-square tests revealed that vaccinated individuals' content of narrative was more positive and less negative than that of unvaccinated individuals (see Table 4). In addition, the vaccinated group experienced more positive, less negative, and less indifferent emotions, while there were no differences in congruence. *T*-tests showed that unvaccinated individuals had higher percentages of certainty as well as mightiness. Figure 2 displays the word clouds of vaccinated vs. unvaccinated individuals, and Table 6 shows the frequencies of the most commonly used words. The most frequent word in both groups was “I”, and the fourth most frequent word in the vaccinated group was “protect”, which was absent in the unvaccinated group. However, the fourth most frequent word in the unvaccinated group was “because”, which came in place 13 in the vaccinated group.

**Table 6:** Word frequencies of the 25 most frequent words for item 2 (“When I think about the COVID-19 vaccination, I believe”) in the Believing Questionnaire for the two groups.

**Vaccinated**				**Unvaccinated**		
Word	Frequency	%^a^		Word	Frequency	%^a^
I	483	6.24		I	76	5.52
Vaccination	308	3.98		Vaccination	55	4.00
Me	217	2.80		Vaccine	43	3.13
Protect	188	2.43		Because	20	1.45
Way	182	2.35		My	17	1.24
My	181	2.34		Me	15	1.09
Course	159	2.05		Can	14	1.02
Pandemic	155	2.00		Which	11	0.80
Can	151	1.95		Still	10	0.73
Severe	151	1.95		Virus	10	0.73
Protects	140	1.81		Approval	9	0.65
From	132	1.71		Risk	9	0.65
Because	124	1.60		Side	9	0.65
Disease	113	1.46		All	8	0.58
Myself	104	1.34		Already	8	0.58
Against	98	1.27		Covid	8	0.58
Protected	77	1.00		Effects	8	0.58
Others	68	0.88		What	8	0.58
Out	67	0.87		Benefit	7	0.51
Helps	65	0.84		From	7	0.51
Life	63	0.81		Person	7	0.51
Infection	62	0.80		Researched	7	0.51
Protection	56	0.72		Tested	7	0.51
Better	55	0.71		Think	7	0.51

^a^Shows what percentage of the total words the word represents.

## Discussion

The topic of “vaccination” was very polarized before the introduction of mandatory vaccination in Austria. As the topic was hotly debated in all areas at the end of 2021, including the media, and WHS are particularly affected by the consequences of the pandemic (1–6), this study aimed to examine the motives and attitudes for the vaccination decision in this group using the credition model by Angel and Seitz (32).

Thus, shortly before the introduction of mandatory COVID-19 vaccination in Austria, 1,062 vaccinated and 97 unvaccinated WHS were surveyed in December 2021/January 2022 about their beliefs regarding the pandemic and the vaccination.

The willingness to vaccinate was high, especially in the male sample (95%). This is in line with other studies showing an association between male sex and a higher willingness to get vaccinated (23, 25, 26). In spite of the majority of participants being female (79.4%), the sample's vaccination rate of 91.6 % was not lower than the vaccination rate of the general population at the time of the online survey [75.9%; (51)]. Moreover, a meta-analysis found a lower vaccination rate in WHS as compared to the general population (24). We assume that our results were mostly determined by the essential sociopolitical conditions in Austria at that time, including the lockdown for unvaccinated people in November 2021, the upcoming mandatory vaccination, and the obligation for unvaccinated WHS to have valid negative COVID-19 tests at work. Although the vaccination was still optional at the time of the survey, all circumstances could have been strong motivators to get vaccinated, leading to a high vaccination rate in WHS. The potential of sampling bias due to the attitude toward vaccination should be considered as well.

Regarding believing processes about the COVID-19 vaccination, both groups reported mostly negative emotions and narratives when asked about the pandemic in general, showing the emotional and mental strain accompanying this global crisis. WHS have been found to have an increased susceptibility to developing mental health problems during the pandemic, as shown by other studies (4, 52). Unvaccinated WHS might be at an even higher risk, as they reported more negative and less positive narratives and emotions in their beliefs than vaccinated WHS. Not only does this finding highlight the relevance and controversy surrounding this topic, but it also shows the abundance of negative feelings of unvaccinated individuals. Perhaps these feelings did not only stem from the mandatory vaccination in particular, but also from the perception that this governmental measure might have been one too many, an infringement upon personal rights that incited an attitude tinged by the thought of rebellion.

In addition, unvaccinated WHS showed higher levels of certainty and mightiness when believing than vaccinated WHS. Especially the latter could be an expression of their insistence on holding on to their belief to not be vaccinated. Despite the increasing pressure from the upcoming mandatory vaccination and the strong negative emotions associated with it, unvaccinated WHS were more confident in their beliefs. This is reminiscent of the phenomenon of justification of effort, a paradigm of cognitive dissonance: people tend to like what they have to work hard for, as opposed to easily achievable goals (53). The high frequency of the word “because” also provides an indication of justification and the search for arguments, more so than in the unvaccinated group, whose opinion was represented by most of the media and experts. Furthermore, unvaccinated WHS might have been influenced by other phenomena of social psychology as well as personal experiences and circumstances.

Vaccinated WHS reported more positive narratives and emotions concerning the vaccination than unvaccinated WHS. Among other possible explanations, one might be provided by the theory of cognitive dissonance (53): firstly, the paradigm of free choice states that cognitive dissonance is created when an individual is faced with the difficult decision to choose between alternatives, which can be influenced by social norms and preferences. After having decided, the chosen option is portrayed as more desirable than the one not chosen to justify one's decision (54, 55), as reflected by vaccinated WHS expressing more positive narratives (57.3%) and emotions (88.9%) in relation to the vaccination than unvaccinated WHS (27.8%, 19.6%). Secondly, according to the induced compliance paradigm, a person forced to say or do something that contradicts their private opinion is inclined to change this opinion or belief (56). At the time of the survey, the vaccination was not yet mandatory, however, participants were aware that it would be in the future, and societal pressure was high regardless. Moreover, as we were strict in using “vaccination yes/no” as the grouping variable and not “immunization yes/no” (≥ 2 shots), the group of vaccinated WHS might have included those who were not entirely content with being vaccinated, but still agreed to do so. Therefore, a positive mental attitude toward the previously undesirable vaccination might have been formed after having received it, as supported by the currently discussed results. Despite the majority of vaccinated WHS experiencing positive emotions (88.9%), our results support the notion that vaccinated WHS have been conflicted nevertheless, as shown by the lower degree of uniformity in content of narratives (57.3%).

Pertaining to word frequencies, the word “vaccination”/”vaccine” stands out as having a particularly high frequency relative to other words used by non-vaccinated WHS when thinking about the vaccination. In combination with “because”, unvaccinated WHS seem to have listed a variety of reasons for not vaccinating. In contrast, most vaccinated WHS were optimistic about the vaccination and emphasized the protection as well as possible avoidance of a severe course of the disease. When comparing word frequencies of item 1 (COVID-19 in general), it becomes apparent that vaccinated WHS used the word “we” more often, showing their focus on community, while unvaccinated WHS may have been more concerned with governmental measures and their impact. As they felt less sufficiently informed about both governmental and workplace-related COVID-19 measures than vaccinated WHS, providing them with adequate information might be reassuring and reduce psychological burden. Moreover, it was shown that vaccine acceptance is positively associated with the perception of being sufficiently educated about COVID-19 (57).

This study had several limitations. First, online studies are prone to sampling bias. Perhaps, motivations to participate were influenced by individuals' attitudes toward the vaccination. Second, due to the Austrian vaccination rate being 75.9% at the time of the survey, the recruitment of vaccinated WHS in a random sample was more likely (51), leading to unequal sample sizes. To survey more unvaccinated individuals, a larger sample size might be considered for further studies. Third, the groups showed sex differences, however, this variable could not be included as a covariate in non-parametric analyses. Fourth, the ability to self-contemplate might have influenced believing processes and the report thereof. Moreover, believing processes could not be examined in their entirety, since only the verbal expressions were evaluated. Fifth, the reduction of qualitative data by using the categories positive, negative, and indifferent was necessary for data analysis. Sixth, believing processes are associated with psychological symptoms and differ between individuals with psychiatric disorders and healthy individuals (58), however, psychological symptoms were not considered in this study. Lastly, we very strictly set the vaccination group variable with “yes” or “no”. Immunization (≥ 2 shots) could also have been used as a group variable, since at the end of 2021, every person in Austria already had the opportunity to be vaccinated twice, however, we wanted to record the extreme opinions and underlying believing processes.

To conclude, unvaccinated WHSs had more negative and less positive thoughts and emotions than vaccinated WHS when thinking about their beliefs concerning the COVID-19 vaccination. Moreover, they were more certain and experienced stronger emotions while believing about both the vaccination and the COVID-19 pandemic in general. Providing unvaccinated WHS with adequate information about the pandemic might be helpful for easing their concerns.

## Data availability statement

The raw data supporting the conclusions of this article will be made available by the authors, without undue reservation.

## Ethics statement

The studies involving human participants were reviewed and approved by Medical University of Graz. The patients/participants provided their written informed consent to participate in this study.

## Author contributions

ND, FF, and ML designed the study. ND and EF performed literature research as well as data analysis and wrote the first draft. FF, ML, LH, NB, JW-S, SB, H-FA, RS, and ER were responsible for proof reading and revising the manuscript. NB was responsible for English proof reading. ES supported the implementation of the study *via* the online application tool LimeSurvey. ER and ND supervised the study procedure and revised important intellectual content. All authors contributed to the article and approved the submitted version.

## Funding

This study was funded by the Land Steiermark.

## Conflict of interest

The authors declare that the research was conducted in the absence of any commercial or financial relationships that could be construed as a potential conflict of interest.

## Publisher's note

All claims expressed in this article are solely those of the authors and do not necessarily represent those of their affiliated organizations, or those of the publisher, the editors and the reviewers. Any product that may be evaluated in this article, or claim that may be made by its manufacturer, is not guaranteed or endorsed by the publisher.
